# Co_0.5_Mn_0.5_Fe_2_O_4_@PMMA Nanoparticles Promotes Preosteoblast Differentiation through Activation of *OPN-BGLAP2-DMP1* Axis and Modulates Osteoclastogenesis under Magnetic Field Conditions

**DOI:** 10.3390/ma14175010

**Published:** 2021-09-02

**Authors:** Krzysztof Marycz, Eliza Turlej, Katarzyna Kornicka-Garbowska, Emilia Zachanowicz, Anna Tomaszewska, Magdalena Kulpa-Greszta, Robert Pązik

**Affiliations:** 1 Department of Experimental Biology, Wrocław University of Environmental and Life Sciences, ul. CK. Norwida 27B, 50-375 Wrocław, Poland; eliza.turlej@upwr.edu.pl (E.T.); katarzyna.kornicka-garbowska@upwr.edu.pl (K.K.-G.); 2 International Institute of Translational Medicine (MIMT), ul. Jesionowa 11, Malin, 55-114 Wisznia Mała, Poland; 3 Polymer Engineering and Technology Division, Wroclaw University of Technology, ul. Wyb. Wyspiańskiego 27, 50-370 Wrocław, Poland; emilia.zachanowicz@pwr.edu.pl; 4 Department of Biotechnology, Institute of Biology and Biotechnology, College of Natural Sciences, University of Rzeszow, Pigonia 1, 35-310 Rzeszow, Poland; atomaszewska@ur.edu.pl (A.T.); mkulpa@ur.edu.pl (M.K.-G.); rpazik@ur.edu.pl (R.P.); 5 Faculty of Chemistry, Rzeszow University of Technology, Aleja Powstańców Warszawy 12, 35-959 Rzeszow, Poland

**Keywords:** osteoporosis, pre-osteoblasts, osteoclasts, PMMA, Co_0.5_Mn_0.5_Fe_2_O_4_, magnetic field, apoptosis, integrins, microRNAs

## Abstract

The prevalence of osteoporosis in recent years is rapidly increasing. For this reason, there is an urgent need to develop bone substitutes and composites able to enhance the regeneration of damaged tissues which meet the patients’ needs. In the case of osteoporosis, personalized, tailored materials should enhance the impaired healing process and restore the balance between osteoblast and osteoclast activity. In this study, we fabricated a novel hybrid material (Co_0.5_Mn_0_._5_Fe_2_O_4_@PMMA) and investigated its properties and potential utility in the treatment of osteoporosis. The material structure was investigated with X-ray diffraction, Fourier-transform infrared spectroscopy with attenuated total reflectance, FTIR-ATR, transmission electron microscopy (TEM), scanning electron microscopy (SEM) and selected area (electron) diffraction (SAED). Then, the biological properties of the material were investigated with pre-osteoblast (MC3T3-E1) and pre-osteoclasts (4B12) and in the presence or absence of magnetic field, using RT-qPCR and RT-PCR. During the studies, we established that the impact of the new hybrids on the pre-osteoblasts and pre-osteoclasts could be modified by the presence of the magnetic field, which could influence on the PMMA covered by magnetic nanoparticles impact on the expression of genes related to the apoptosis, cells differentiation, adhesion, microRNAs or regulating the inflammatory processes in both murine cell lines. In summary, the Co_0.5_Mn_0_._5_Fe_2_O_4_@PMMA hybrid may represent a novel approach for material optimization and may be a way forward in the fabrication of scaffolds with enhanced bioactivity that benefits osteoporotic patients.

## 1. Introduction

Osteoporosis (OP) is to the most frequent bone disease which can occur at any age stage, although women are more predisposed than men [1]. OP is common in societies all over the world due to progressive aging. The disease is characterized by reduced bone strength, mineral density and biomechanical properties, which together trigger bone fractures. According to the NIH Consensus Development Panel on Osteoporosis Prevention, osteoporosis is defined to “primarily reflect the integration of bone density and bone quality [2]. OP related bone fracture seriously affects patients’ quality of life, with increased mortality and disability. More than 50% of postmenopausal women suffer from bone fracture and less than 30% of them will recover to normal life after hip fracture. Although the incidents of OP among men are estimated to be around 20%, mortalities due to bone fractures including the hip are twice as frequent in women [3]. Together with progressing aging in society, it is easily predicted that OP-related bone fracture incidents will steadily grow in the future; therefore, further research searching for factors that could improve bone regeneration are strongly required and fully reasonable. The bone microstructure under OP condition is seriously deteriorated and decreased bone mineral density (BMD), causing abnormal bone remodeling in which the advantage of anabolic process over catabolic occurs. This process is mediated by two critical cell populations, i.e., osteoblasts that are responsible for the synthesis and secretion of multiplate factors leading to bone formation and osteoclasts that resorb the bone. Osteoblasts originate from multipotent stem progenitor cells that give them rise and are critical for the development of functionally active osteoblasts. These multipotent stem progenitor cells, apart from osteoblasts, give rise to other adult cell populations including chondrocytes, adipocytes or myocytes underlining their importance during tissue formation [4]. Within osteoblasts, runt-related transcription factor 2 (RUNX2), bone γ-carboxyglutamate protein 2 (Bglap2) and dentin matrix protein 1 (Dmp1) are recognized as a critical regulator of osteoblast differentiation and function. Osteoblast formation, function and proliferative activity are mediated by multiplate signaling pathways and proteins including TGF-β/BMP and WNT pathways and osteopontin (OPN), which modulates the expression of common transcripts involved in the bone formation process [5]. OPN plays an important role in bone metabolism and homeostasis, especially in endocrine-regulated bone mass, osteoblast proliferation, migration and adhesion. OPN has been proposed in several studies to be closely related to the development of many bone diseases including osteosarcoma and osteoporosis as its impact on the bone formation/resorption process seems to be critical [6]. In turn, bone resorption is critically regulated by osteoclasts which are cells differentiated from the myeloid precursors. The activity of osteoclasts is mediated by various factors including cell-to-cell contact (osteoblast-osteoclasts), secretion of a broad range of cytokines colony-stimulating factor 1 (CSF1), receptor activator of NF-κB ligand (RANKL) that activate signaling pathways in myeloid precursors leading to enhanced expression of transcription factors critical for osteoclasts differentiation including PU.1, cFOS and NF-κB [7]. Pu.1 seems to play a critical role since its depletion in mouse model directly leads to osteoporosis development. Pu.1 is the master regulator of hematopoietic precursors to the myeloid lineage and it has been shown to be a critical activator of osteoclast differentiation [8].

Interest in organic-inorganic hybrid materials is caused by the possibility of mixing different material properties into one single product to deliver smart and multifunctional platforms for broad biomedical applications. In the case of ferrite compounds, the tailoring of the elemental composition of magnetic spinal leads to the mixed character of magnetic properties, i.e., hard–soft behavior that enhances their response to the applied magnetic field and increases the efficacy of temperature generation in hyperthermia [9]. The addition of the polymeric outer layer to the inorganic core improves the biocompatibility by protecting particle surfaces from contact with biological objects. Among many accessible polymers, polymethacrylate (PMMA) deserves special attention since it is used as the main component of injectable medical cement and a popular filling material characterized at the same time with very low toxicity [10]. The role of hyamine 1622 (benzethonium chloride) is essential since except for being a cationic surfactant, it can deliver anticancer and antiviral activity to organic-inorganic hybrid materials [11]. Recently, it was shown that this type of organic-magnetic hybrid materials has great potential in the treatment of deep-seated cancer, as well as being important composites in a new class of bone replacement materials for temperature stimulation of regenerative processes [12,13].

For that reason, in the presented study, we developed Co_0.5_Mn_0.5_Fe_2_O_4_@PMMA nanohybrids that were cultured with both pre-osteoblasts and osteoclasts under normal and static magnetic field conditions. In this study, we have found that under normal and magnetic field condition, Co_0.5_Mn_0.5_Fe_2_O_4_@PMMA improves pre-osteoblast activity and induces the expression of *OPN-BGLAP2-DMP1* axis by activation of integrins, while inhibiting osteoclastogenesis.

In summary, by utilizing Co_0.5_Mn_0.5_Fe_2_O_4_@PMMA nanohybrids modulating of osteoblasts/osteoclast activity might occur, therefore becoming an interesting approach for developing a strategy for future osteoporotic related fracture and bone regeneration. 

## 2. Materials and Methods

### 2.1. In Situ Synthesis of the Binary Co_0.5_Mn_0.5_Fe_2_O_4_@PMMA Hybrids

The magnetic field responsive stock colloidal suspension of the Co_0.5_Mn_0.5_Fe_2_O_4_ nanoparticles was prepared via microwave driven non-hydrolytic approach described in detail elsewhere [14]. The magnetic characterization of Co_0.5_Mn_0.5_Fe_2_O_4_ was presented by our group previously interested reader is advised to follow that article [14]. In short, the following metal acetylacetonates were taken 1 mmol (257 mg) of Co(acac)_2_ (99.9%, Alfa Aesar, Kandel, Germany), 1 mmol (253 mg) of Mn(acac)_2_ (99.9% Alfa Aesar, Kandel Germany), 4 mmol (1413 mg) of Fe(acac)_3_ (99.99%, Alfa Aesar, Kandel Germany) and subsequently dissolved in 70 mL of acetophenone (99% Sigma Aldrich, Poznań, Poland, used without further purification). All handling with the metal complexes has been done under an inert atmosphere of N_2_ using an acrylic glove box (GS Glove Box Systemtechnik GMBH P10R250T2, Sömmerda, Germany). The reaction mixture was directly transferred into the Teflon vessel, secured and placed inside of the Ertec^®^ Magnum V2 microwave reactor (Ertec, Wrocław, Poland). The process was carried out under autogenous pressure of 15 atm, at 200 °C for 60 min. Afterward, Co_0.5_Mn_0.5_Fe_2_O_4_ nanoparticles were separated from the solvent through washing, centrifuging cycles and re-suspended in 30 mL of de-ionized water. The final concentration of the Co_0.5_Mn_0.5_Fe_2_O_4_ particles was measured using the micro-scale technique. In the case of the binary Co_0.5_Mn_0.5_Fe_2_O_4_@PMMA hybrids, the in situ polymerization protocol was adopted without any changes as described previously [15]. The main chemicals were methyl methacrylate (MMA) monomer (99% Sigma Aldrich, Poznań, Poland), potassium peroxydisulfate as a polymerization initiator (≥99.0%, KPS, Sigma Aldrich, Poznań, Poland), benzethonium chloride (≥96.0%, Hyamine^®^1622, Sigma Aldrich, Poznań, Poland), as well as stock Co_0.5_Mn_0.5_Fe_2_O_4_ particles. The MMA monomer was purified from the hydroquinone (inhibitor) through washing with 10% water solution of NaOH (≥97.0% Sigma Aldrich, Poznań, Poland) and, finally, dried with MgSO_4_ (≥97.0% Sigma Aldrich, Poznań, Poland) prior usage. Briefly, 3 mL (4 mmol) of hyamine containing aqueous solution was added to the 6 mL of the Co_0.5_Mn_0.5_Fe_2_O_4_ (concentrated stock suspension containing 100 mg particles) and mixed with 13 mL of de-ionized water. An ultrasound bath was used to homogenize dispersion for 20 min. After that, the mixture was transferred to a four-neck flask equipped with a mechanical stirrer, gas inlet (N_2_), dropping funnels and Pt-100 sensor for temperature control. The MMA (in proportion of 80% to 20% of particles) was slowly injected and a KPS initiator was added. The reaction vessel was heated up to 80 °C for 3 h under constant stirring and nitrogen blanket. The binary hybrids were separated using a laboratory magnet and carefully dried under a vacuum.

### 2.2. Characterization of Basic Physicochemical Properties of PMMA@Co_0.5_Mn_0.5_Fe_2_O_4_ Hybrids

Structure identification of Co_0.5_Mn_0.5_Fe_2_O_4_ nanoparticles and Co_0.5_Mn_0.5_Fe_2_O_4_@PMMA hybrid materials was carried out employing X-ray powder diffraction technique using a PANalytical X’Pert PRO X-ray diffractometer (Cu-*K*_α1_ = 1.54060 Å, nickel filtering, Malvern, UK) recording XRD patterns at the range of 2Q = 10–75° and their direct comparison with the reference standards from the ICDD database (International Centre for Diffraction Data). The morphology and particle size were discussed based on the transmission electron microscopy (TEM) in the case of the nanoparticles whereas hybrid materials were subjected to the scanning electron microscopy (SEM) technique to prevent hybrids from unwanted deterioration induced by the high energy electron beam. Therefore, a Philips CM-20 Super Twin microscope (Philips, Amsterdam, The Netherlands), operated at 200 kV was used for the TEM characterization while hybrids samples were imaged with a Nova Nano-SEM 230 microscope (FEI Company, ThermoFisher Scienitific, Waltham, MA, USA). The measurements of the FTIR-ATR spectra were performed on a Nicolet iZ10 spectrometer (Thermo Fischer Scientific, Waltham, MA, USA) equipped in diamond ATR accessory covering the spectral range between 4000–500 cm^−1^ at room temperature. Magnetic characterization of the Co_1−x_Mn_x_Fe_2_O_4_ colloids was presented by us previously [16]; thus, the interested reader is advised to follow that article for details. The hybrid particle mean size and distribution were estimated by using free-image processing software ImageJ v.1.46 [17] through analysis of SEM images by taking into consideration of 100 hybrid particles (longest diameter was measured due to elongated shape of objects).

### 2.3. Cell Lines

The mouse pre-osteoblast mouse cell line (MC3T3-E1-subclone 4) was obtained from the European Collection of Authenticated Cell Cultures (EACC, Sigma-Aldrich, Munich, Germany), while the mouse pre-osteoclast cell line (4B12) was a kind gift from the Department of Oral Biology and Tissue Engineering, Meikai University School of Dentistry (Professor S. Amano) [18]. The MC3T3-E1 cell line was maintained in Minimum Essential Medium Alpha (MEM-α, Gibco, Waltham, MA, USA) without ascorbic acid supplemented with 10% Fetal Bovine Serum (FBS, Merck, KGaA, Darmstadt, Germany) and 1% of standard antibiotics (Merck KGaA, Darmstadt, Germany). The 4B12 cell line was also cultured in MEM-α with the addition of 30% CSCM (calvaria-derived stromal cell conditioned media), 10% of FBS and 1% of antibiotics in standard conditions.

### 2.4. Magnetic Field (MF)

**Figure 1:**
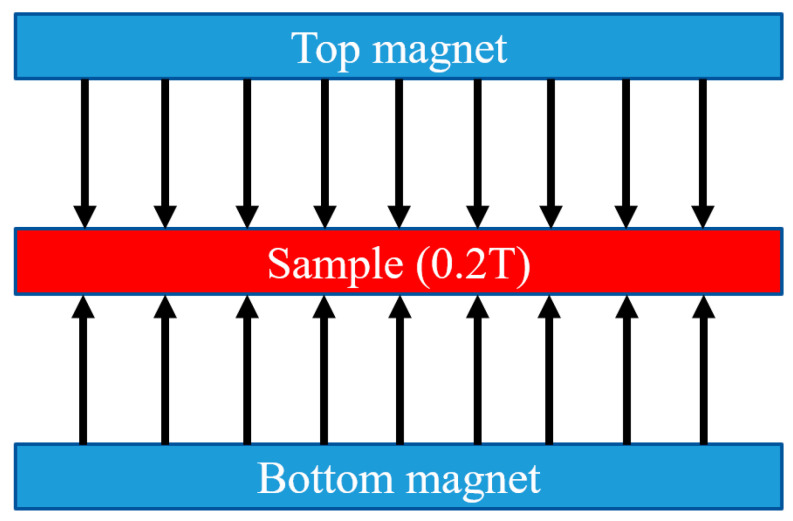
Diagram of the magnetic field.

The cells were exposed to the magnetic field using the static magnetic field (SMF) stimulation system designed in Wroclaw University of Science and Technology. This system is appropriate to produce a uniform SMF through action of two parallel magnets with opposite polarity. Plates with samples were placed in the central core, as shown in Figure 1. In this place, the MF strength was equaled 0.3T. The daily exposure of cells on the MF was 15 min.

### 2.5. Cell Proliferation Assay

In order to determine the viability of cells after treating them with PMMA, Co_0.5_Mn_0.5_Fe_2_O_4_ and their combination (Co_0.5_Mn_0.5_Fe_2_O_4_@PMMA) in a ratio of 80/20 and at a final concentration of 90.8 μg/mL, after 24, 48 and 72 h in the magnetic field conditions, TOX-8 kit (Merck KGaA, Darmstadt, Germany) was performed according to the manufacturer’s protocol. The absorbance in the appropriate wells was evaluated by 96-well microplate reader (Epoch; Biotek Instruments, Winnoski, VT, USA) equipped with Gen5 software version 2.0 [19]. The measurements were taken at 600 nm and 690 nm as the reference lengths. Each experiment was performed at least three times independently. 

### 2.6. Morphology and Mitochondria Status Analysis

The mitochondria, actin filaments and the nucleus of treated/untreated pre-osteoblasts and pre-osteoclasts were stained as described previously [20]. Briefly, mitochondria were stained using MitoRed dye, the F-actin filaments with Phalloidin-Atto 488 and the cells nuclei with 4′,6-diamidino-2-phenylindole DAPI (all from Life Technologies, Carslbad, CA, USA). 

Briefly, the cells after incubation with the PMMA and its combination, were incubated for 30 min with MitoRed solution at concentration 1:1000 at 37 °C and fixed with 4% PFA (POCh, Gliwice, Poland). Then, the cells were stained with Phalloidin-Atto 488 for 45 min at RT and then stained with DAPI. Visualization was made by a confocal microscope (Leica TCS SPE, Leica Microsystems, Wetzlar, Germany) at 0.5 µm steps up to a final depth of 25 µm. Images were captured at magnification 630× and analyzed using Fiji New ImageJ with Colour Pixel Counter plugin version 1.52 developed by Wayne Rasband from NIH, USA. Each photograph was taken at least three times independently.

### 2.7. Gene Expression Analysis

The gene expression analysis was performed using qPCR technique. Briefly, cells seeded at the plastic plates at the density of 1 × 10^4^/well in the appropriate medium with the addition of PMMA or its modification and placed in the magnetic field were collected after 24 h and suspended in the Extrazol (BLIRT DNA, Gdańsk, Poland). The total RNA was obtained using acid guanidinium thiocyanate-phenol-chloroform extraction method described by Chomczynski and Sacchi [21] using the reagents from Merck KGaA, Darmstadt, Germany. The total of RNA quality and quantity were determined using a spectrophotometer (Epoch, Biotek Instruments, Winnoski, VT, USA). 

The process of digestion of gDNA and cDNA synthesis was performed using Takara PrimeScript™ RT Reagent Kit with gDNA Eraser (Perfect Real Time) (Takara, Bio Europe, Goteborg, Sweden) according to the manufacturer protocol. Both processes were performed using a T100 Thermal Cycler (Bio-Rad, Hercules, CA, USA). 

Each cDNA template was amplified by the quantitative reverse transcription polymerase chain reaction, using SensiFAST™ SYBR No-ROX Kit (Bioline, London, UK) in total volume of 10 µL (for a single reaction-1 μL of cDNA and 500 nM of each primer, according to the protocol). The sequences of the specific primers obtained from Merck KGaA, are listed in Table 1. The qRT-PCR reactions were performed using a CFX Connect Real-Time PCR Detection System (CFX Connect Optics Module, Bio-Rad, Hercules, CA, USA) equipped with the software BioRad CFX Maestro and the transcript levels were normalized to *Gaph* as a standard control (house-keeping gene).

### 2.8. Statistical Analysis

Statistical analysis was performed using GraphPad Prism 5 Software and the statistical significance was marked with asterisk (*). The *p* value less than 0.05 (*p* < 0.05) are marked with one asterisk (*), while *p* value less than 0.01 (*p* < 0.01) with two asterisks (**) and, finally, the *p* values less than 0.001 (*p* < 0.001) with three asterisks (***).

## 3. Results

### 3.1. Characterization of Physicochemical Properties of the Co_0.5_Mn_0.5_Fe_2_O_4_ Nanoparticles and PMMA@Co_0.5_Mn_0.5_Fe_2_O_4_

**Figure 2:**
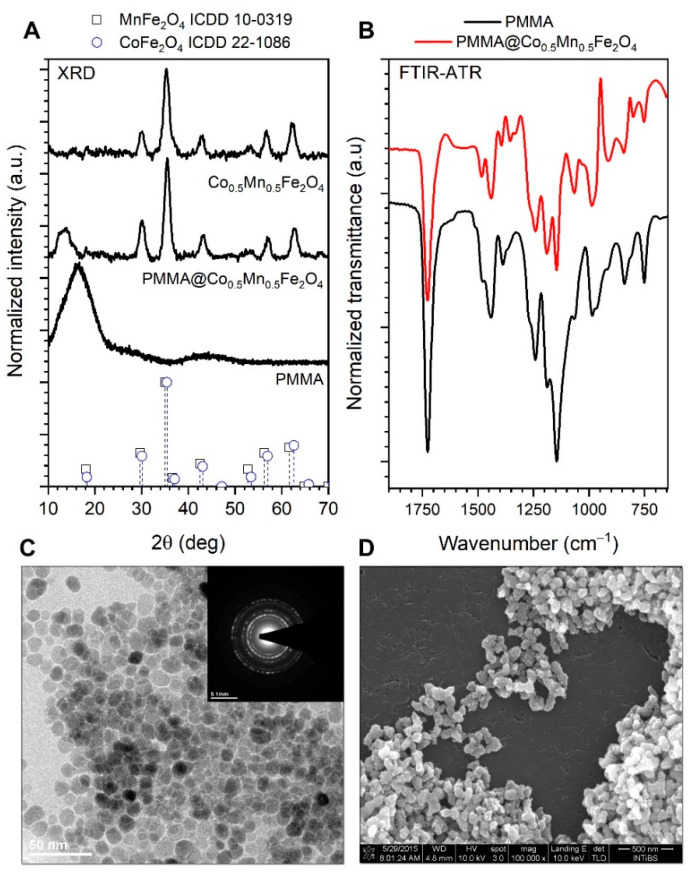
X-ray diffraction patterns of the Co_0.5_Mn_0.5_Fe_2_O_4_ and Co_0.5_Mn_0.5_Fe_2_O_4_@PMMA (**A**,**B**) FTIR-ATR spectra of the reference PMMA and composite sample; (**C**) TEM and SAED images of the Co_0.5_Mn_0.5_Fe_2_O_4_ nanoparticles, as well as (**D**) SEM picture of the binary hybrid.

The analysis of the diffraction patterns (Figure 2A) leads to the conclusion that the structure of the Co_0.5_Mn_0.5_Fe_2_O_4_ can be ascribed to the spinel-type materials as supported by reference card no. ICDD 10–0319 as well as 22–1086. A detailed analysis of the structural properties of the same nanoparticles with a broader concentration range of Mn doping was a subject of our previous article [15] where it was proven that the final compound formed solid solution of respective elements in appropriate ratio. The results of the formation of the hybrid material were confirmed by the FTIR-ATR spectra analysis (Figure 2B). One can see a range of the PMMA characteristic vibration modes which are present either in the case of hybrid Co_0.5_Mn_0.5_Fe_2_O_4_@PMMA and reference polymer sample prepared in the same way as composite. The difference in peak positions and their structure reflects the interaction of the PMMA with the Co_0.5_Mn_0.5_Fe_2_O_4_ surface [22]. The average crystallite size was calculated from the peak broadening with help of Scherrer’s formula:

(1) $$D=\frac{k\lambda }{cos\theta \sqrt{\beta^{2}-\beta_{0}^{2}}}$$

where *k* stands for constant value set at 0.9, *λ* is the wavelength of the Cu lamp (1.54060 Å), *β*_0_ means apparatus broadening; *β* is full width at half maximum (FWHM) and θ corresponds with the peak maximum taken for the calculations [22] and compared with the size extracted from the TEM image (Figure 2C). We noted that there is a very good match between both size estimation methods. The mean crystallite size calculated from the broadening of the (220) crystallographic plane reflection was around 7 nm, whereas the particle size obtained from the TEM imaging is 8 ± 2 nm. Analysis of the SEM micrographs leads to the observation that the hybrid materials have elongated shapes, sample morphology shows sufficient homogeneity, while the size of the objects has been estimated to range from 120 to 200 nm. 

All reflections were indexed accordingly; the 14 2θ broad peak corresponds to the amorphous PMMA shell.

The Co_0.5_Mn_0.5_Fe_2_O_4_ sample was taken as a core material due to its best magnetic properties within the whole concentration range of Mn^2+^ studied in Ref. [14]. The sample and the sample after covering it with the PMMA shell (to improve biocompatibility) assure the best response upon action of static magnetic field. 

### 3.2. Anti-Proliferative Effect of PMMA Modified by Co_0.5_Mn_0.5_Fe_2_O_4_ in Ratio 80/20 towards Pre-Osteoblasts and Pre-Osteoclasts in the Presence of Magnetic Field

**Figure 3:**
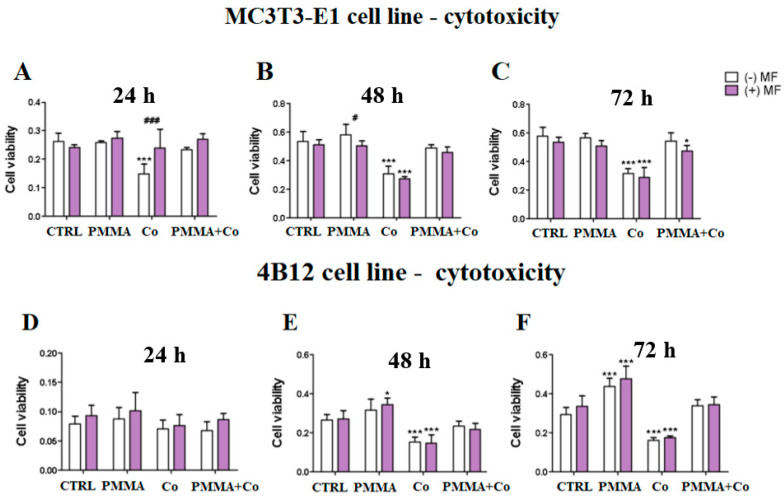
The kinetics of anti-proliferative effects of PMMA and PMMA@Co_0.5_Mn_0.5_Fe_2_O_4_ towards mouse pre-osteoblasts (MC3T3-E1 cell line) after 24 h (**A**), 48 h (**B**) and 72 h (**C**) and towards mouse pre-osteoclasts (4B12 cell line) after 24 h (**D**), 48 h (**E**) and 72 h (**F**) in the presence of the magnetic field. Notes: CTRL—control, PMMA—poly(methyl (methylacrylate), Co—Co_0.5_Mn_0.5_Fe_2_O_4_, PMMA + Co—Co_0.5_Mn_0.5_Fe_2_O_4_@PMMA in ratio 80/20, MF—magnetic field. Statistical differences are indicated by * *p* < 0.005 and *** *p* < 0.0001 (in comparison to control) and by ^#^ *p* < 0.005; ^###^ *p* < 0.0001 (comparison MF+/MF−).

The effect of PMMA and Co_0.5_Mn_0.5_Fe_2_O_4_@PMMA modification was evaluated on the pre-osteoblasts in the presence of magnetic field and the results showed that applying of magnetic fields decrease the viability of MC3T3-E1 after 48 h (Figure 3B). 

In turn, Co_0.5_Mn_0.5_Fe_2_O_4_@PMMA increases the viability in the MF(+) condition in comparison to control cells (Figure 3C).

In the case of pre-osteoclasts, we observed statistically significant increase of viability of cells treated with the PMMA in the presence of MF(+) after 48 h (Figure 3E) and after 72 h, independently of the MF application (Figure 3F)

### 3.3. Morphology and Mitochondria Network Development Related to PMMA and PMMA@Co_0.5_Mn_0.5_Fe_2_O_4_ towards Osteoblasts and Osteoclasts in the Presence of Magnetic Field

The impact of the PMMA and its modification on the morphology and mitochondria network rearrangement in the magnetic field on the pre-osteoblasts and pre-osteoclasts was determined using MitoRed and F-actin staining.

**Figure 4:**
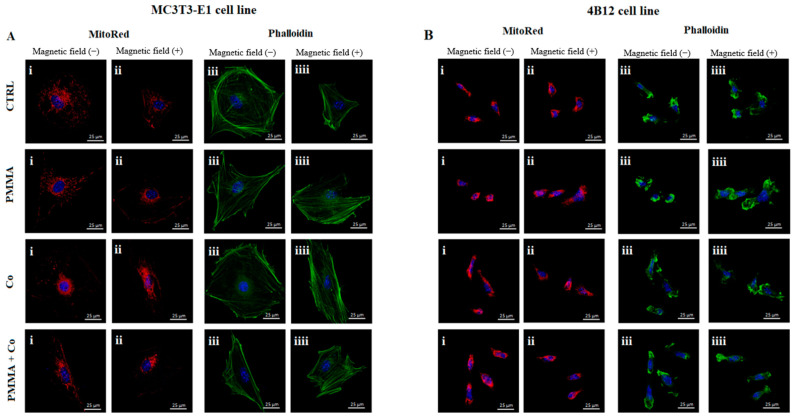
The impact of the PMMA and its modification on the mitochondria status and cytoskeleton in the MC3T3-E1 (**A**) and 4B12 (**B**) cell line in the presence of the magnetic field. Notes: CTRL—control, PMMA—poly(methyl (methylacrylate)), Co—Co_0.5_Mn_0.5_Fe_2_O_4_, PMMA+ Co—Co_0.5_Mn_0.5_Fe_2_O_4_@PMMA in ratio 80/20.

Our studies showed that PMMA alone Co_0.5_Mn_0.5_Fe_2_O_4_@PMMA limited the growth of cytoskeleton in the pre-osteoblasts in the MF(−) condition (Figure 4A(iii)). Moreover, the comparison between MF(+) and MF(−) revealed that pre-osteoblasts cultured in the MF(+) condition showed weak cytoskeleton development than these in the MF(−) (Figure 4A(iii,iiii)). From the other side, the mitochondrial network was less developed in case of PMMA combination in the MF(−) condition in comparison to control cells (Figure 4A(i)).

The opposite effect was reported in the pre-osteoclasts treated by PMMA and its combination in both magnetic conditions, where the clearly visible mitochondrial network and cytoskeleton development were reported (Figure 4B(i–iiii)).

The photographs were captured at 60× magnification: scale bar, 25 μm. 

### 3.4. The Impact of PMMA Modified by Co_0.5_Mn_0.5_Fe_2_O_4_ in Ratio 80/20 on the Expression of Genes Related to the Apoptosis towards Pre-Osteoblasts and Pre-Osteoclasts in the Presence of Magnetic Field

The impact of the PMMA and its modification on the apoptosis of pre-osteoblasts and pre-osteoclasts was determined using qPCR. The expression of *p21*, *p53*, *Casp9*, *Bad*, *Bax* and *Bcl-2* was analyzed after 24 h of incubation of the cells with the addition of PMMA and Co_0.5_Mn_0.5_Fe_2_O_4_@PMMA in ratio 80/20 in the MF condition. 

**Figure 5:**
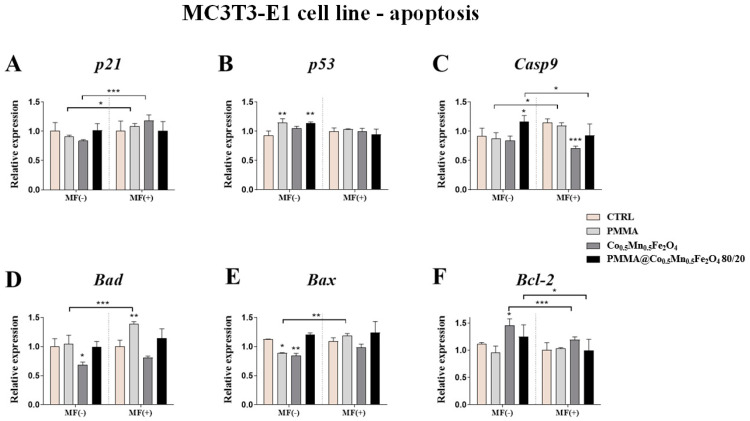
The impact of PMMA and its modification on the expression of genes related to apoptosis: (*p21* (**A**), *p53* (**B**), *Casp-9* (**C**), *Bad* (**D**), *Bax* (**E**) and *Bcl-2* (**F**)) in the magnetic field condition towards mouse pre-osteoblasts (MC3T3-E1 cell line). Statistical differences are indicated by * *p* < 0.005; ** *p* < 0.001 and *** *p* < 0.0001.

Our studies showed that PMMA alone increasing the expression of *p53* gene as compared to control in the MF(−) condition (Figure 5B). Moreover, the effect of the magnetic field application was observed after pre-osteoblasts culturing with PMMA. The addition of PMMA caused increase of the expression of the *p21*, *Casp9*, *Bad* and *Bax* in the MF(+) as compared to MF(−) (Figure 5A,C–E). 

In turn, the expression of the *p53* gene after 24 h exposition to Co_0.5_Mn_0.5_Fe_2_O_4_@PMMA was increased in the MF(−) condition, compared to control (Figure 5B). In parallel, the expression of the *Casp9* and *Bcl-2* were increased after PMMA@Co_0.5_Mn_0.5_Fe_2_O_4_ incubation in the MF(−), in comparison to MF(+) (Figure 5C,F).

**Figure 6:**
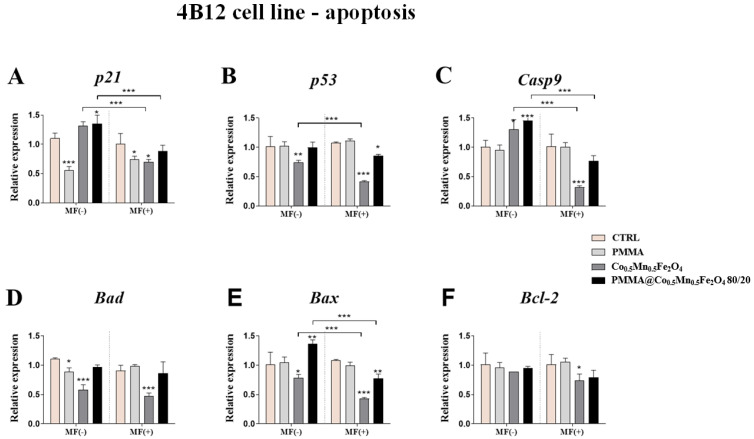
The impact of PMMA and its modification on the expression of genes related to apoptosis: (*p21* (**A**), *p53* (**B**), *Casp-9* (**C**), *Bad* (**D**), *Bax* (**E**) and *Bcl-2* (**F**)) in the magnetic field condition towards mouse pre-osteoclasts (4B12 cell line). Statistical differences are indicated by * *p* < 0.005; ** *p* < 0.001 and *** *p* < 0.0001.

The effect of PMMA alone on the pre-osteoclasts expression of genes associated with apoptosis was similar in the MF(+) and MF(−) conditions. The significant decrease in the expression of *p21* and *Bad* in the MF(−) condition, as well as the decrease of the expression of *p21* in the MF(+) condition, was observed (Figure 6A,D).

The opposite effect was observed after pre-osteoclasts treating with Co_0.5_Mn_0.5_Fe_2_O_4_@PMMA in ratio 80/20 in the MF(−) condition, where we observed the increase of the expression of the *p21*, *Casp9* and *Bax* (Figure 6A,C,E), while after the applied magnetic field, the significant decrease of *p53* and *Bax* was reported (Figure 6B,E). Interestingly, applying of magnetic field decreased the expression of *p21*, *Casp9* and *Bax* after treating pre-osteoclasts with modified PMMA (Figure 6A,C,E).

### 3.5. The Impact of PMMA Modified by Co_0.5_Mn_0.5_Fe_2_O_4_ in Ratio 80/20 on the Expression of Genes and Proteins Related to Osteogenesis/Osteoclastogenesiss towards Pre-Osteoblasts and Pre-Osteoclasts in the Presence of Magnetic Field

**Figure 7:**
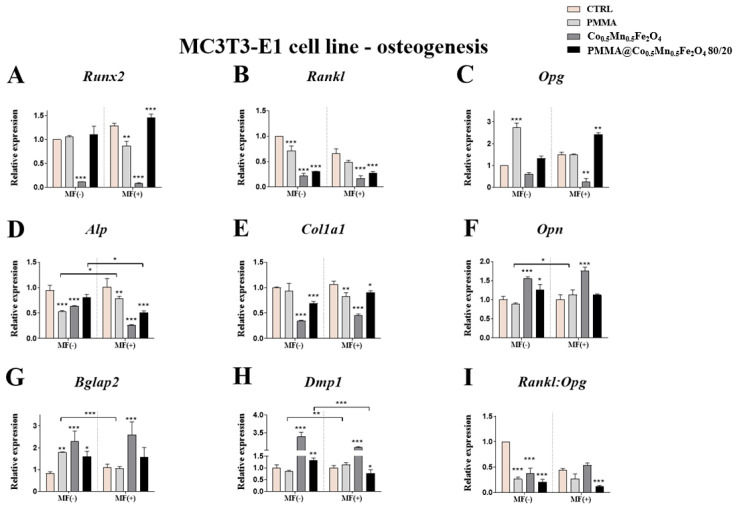
The impact of PMMA and its modification on the expression of genes related to osteogenesis: *Runx2* (**A**), *Rankl* (**B**), *Opg* (**C**), *Alp* (**D**), *Col1A1* (**E**), *Opn* (**F**), *Bglap2* (**G**), *Dmp1* (**H**), *Rankl:Opg* (**I**) in the magnetic field condition towards mouse pre-osteoblasts (MC3T3-E1 cell line).Statistical differences are indicated by * *p* < 0.005; ** *p* < 0.001 and *** *p* < 0.0001.

Our studies revealed the impact of the PMMA on the expression of genes related to process of osteogenesis and osteoclastogenesis in the case of the exposition of them to the magnetic field. Interesting observation was noticed in case of the expression of *Alp* gene after pre-osteoblasts incubation with PMMA. From one side, the expression of the *Alp* was decreased independently of MF application, but from the other side, its expression was lower in the MF(−) than in MF(+) condition (Figure 7B). Moreover, PMMA caused the statistical significant decrease of the *Col1A1* expression in the MF(+) condition in comparison to control (Figure 7C).

The impact of the magnetic field application was also observed in the case of *Bglap 2* and *Dmp1* expression after PMMA addition to culture. In the MF(−) condition, the expression of *Bglap 2* was increased as compared to control, while after comparison *Bglap2* expression between MF(−) and MF(+), we noticed the increase expression after PMMA in the MF(−) than in MF(+) (Figure 7E). Moreover, the expression of *Dmp1* in the MF(+) was higher than in the MF(−) condition (Figure 7F).

In turn, the combination of the Co_0.5_Mn_0.5_Fe_2_O_4_@PMMA increased the expression of *Alp* in the pre-osteoblasts in MF(+) as compared to MF(−) condition (Figure 7B), while *Opn* and *Bglap 2* was increased as compared to the control only in the case of the MF(−) condition (Figure 7D,E). Additionally, the combination of the Co_0.5_Mn_0.5_Fe_2_O_4_@PMMA in a ratio of 80/20 increases the expression of the *Col1A1* as compared to control in both magnetic conditions (Figure 7C).

Interestingly, the expression of the *Dmp1* was increased in the MF(−) condition as compared to control, while in the MF(+) was decreased. Moreover, the expression of the *Dmp1* in the MF(−) condition was increased as compared to MF(+) (Figure 7F).

The impact of the PMMA and its combination in the magnetic field condition on the pre-osteoclasts was determined based on the expression of the *Mmp9, PU.1, Itgav* and *c-fos.*

**Figure 8:**
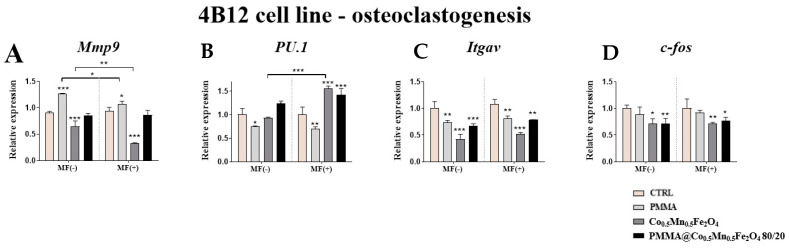
The impact of PMMA and its modification on the expression of genes related to osteoclastogenesis; *Mmp9* (**A**), *PU.1* (**B**), *Itgav* (**C**), *c-fos* (**D**) in the magnetic field condition towards mouse pre-osteoclasts (4B12 cell line). Statistical differences are indicates by * *p* < 0.005; ** *p* < 0.001 and *** *p* < 0.0001.

Our studies revealed the enhanced expression of the *Mmp9* after PMMA incubation of pre-osteoclasts as compared to control cells independently of the magnetic field conditions (Figure 8A). In addition, we reported that presence of the magnetic field is associated with decreasing expression of *Mmp9* (Figure 8A). Additionally, it was noticed that in both magnetic conditions the expression of *PU.1* and *Itgav* was decreased as compared to control (Figure 8B,C).

Similar effect we observed after treating osteoclasts with the combination of Co_0.5_Mn_0.5_Fe_2_O_4_ and PMMA, where the expression of the *PU.1* was increased, while *Itgav* and *c-fos* decreased as compared to control in both magnetic conditions (Figure 8B–D).

### 3.6. The Impact of PMMA Modified by Co_0.5_Mn_0.5_Fe_2_O_4_ in Ratio 80/20 on the Expression of Genes Related to Integrins towards Pre-Osteoblasts and Pre-Osteoclasts in the Presence of Magnetic Field

**Figure 9:**
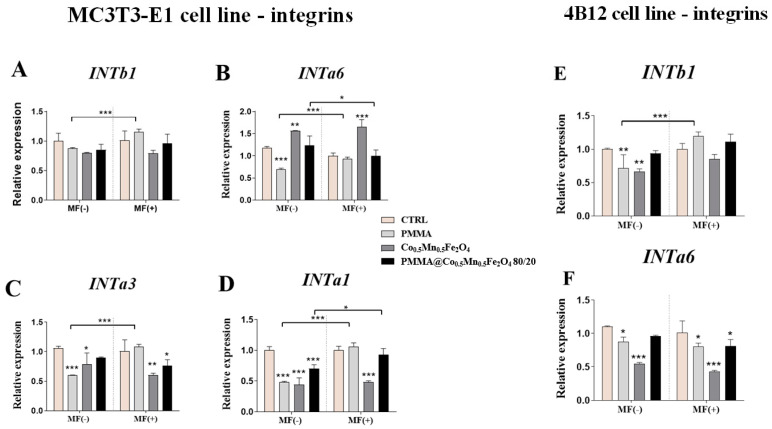
The impact of PMMA and its modification on the integrins expression in the magnetic field condition towards mouse pre-osteoblasts (MC3T3-E1 cell line) *Intb1* (**A**), *Inta6* (**B**), *Inta1* (**C**), *Inta3* (**D**) and mouse pre-osteoclasts (4B12 cell line) *Intb1* (**E**), *Inta6* (**F**). Notes: CTRL—control, PMMA—poly(methyl (methylacrylate)), Co—Co_0.5_Mn_0.5_Fe_2_O_4_, PMMA+ Co—Co_0.5_Mn_0.5_Fe_2_O_4_@PMMA in ratio 80/20. Statistical differences are indicated by * *p* < 0.005; ** *p* < 0.001 and *** *p* < 0.0001.

Finally, we established the impact of the PMMA and its modification on the integrins expression in mouse pre-osteoblasts and pre-osteoclasts cell lines. In the case of *INTb1*, *INTa1*, *INTa3* and *INTa6*, the increase of expression was observed in the MF(+) condition as compared to MF(−) (Figure 9A–D). Moreover, in the absence of magnetic field, the decrease of the expression of the *INTa6*, *INTa1* and *INTaα3* after PMMA culturing of the pre-osteoblasts was observed in the MF(−) conditions (Figure 9B–D). 

In turn, the combination of the PMMA and Co_0.5_Mn_0.5_Fe_2_O_4_@PMMA decreased the expression of the *INTa1* in the MF(−) condition as well as *INTa3* in the MF(+) condition in pre-osteoblasts (Figure 9C,D). Additionally, the impact of the magnetic field on the expression of *INTa6* and *INTa3* after this combination was reported, although in case of the *INTa6* the expression was decreased in the MF(+) condition as compared to MF(−), while in case of the *INTa3*, it was increased in the MF(+) condition (Figure 9B,D).

In the case of the pre-osteoclasts, PMMA alone decreased the expression of the *INTb1* in the presence of the magnetic field and also *INTa6* in both magnetic conditions (Figure 9E,F). Moreover, the combination of the PMMA and Co_0.5_Mn_0.5_Fe_2_O_4_ decrease the expression of the *INTa6* in the magnetic field condition (Figure 9F). Interestingly, the expression of the *INTb1* was increased in the pre-osteoclasts in the MF(+) condition after culturing them in the presence of the PMMA combination.

### 3.7. The Impact of PMMA Modified by Co_0.5_Mn_0.5_Fe_2_O_4_ in Ratio 80/20 on the Expression of Genes Related to Inflammation Process towards Pre-Osteoblasts and Pre-Osteoclasts in the Presence of Magnetic Field

**Figure 10:**
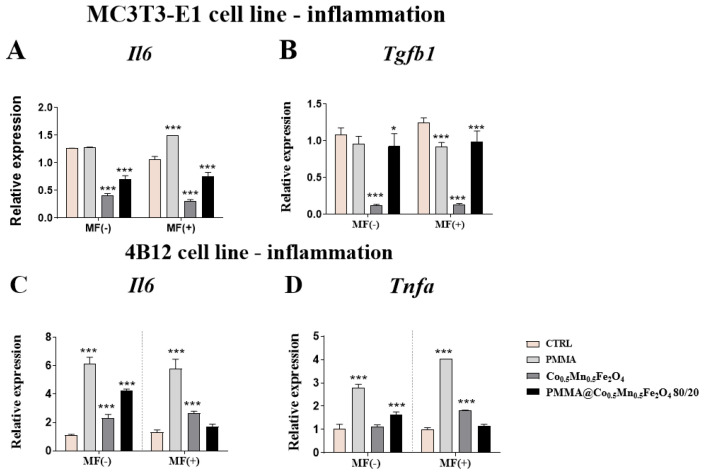
The impact of PMMA and its modification on the expression of genes related to inflammation profile under the magnetic field condition towards mouse pre-osteoblasts (MC3T3-E1 cell line) *Il-6* (**A**), *Tgfb1* (**B**) and mouse pre-osteoclasts (4B12 cell line) *Il-6* (**C**) and *Tnfa* (**D**). Statistical differences are indicated by * *p* < 0.005; and *** *p* < 0.0001.

Interestingly, the results associated with the inflammation profile showed that the new modification of PMMA could decrease the expression of *Il-6* independently of the presence of magnetic field in the pre-osteoblasts, while increasing in the pre-osteoclasts (Figure 10A,C). The opposite, also independently of the presence of MF, Co_0.5_Mn_0.5_Fe_2_O_4_ @PMMA in a ratio of 80/20 caused the decrease of the *Tgfb* in MC3T3-E1 cell line and enhanced the expression of *Tnfa* (Figure 10B,D).

### 3.8. The Impact of PMMA Modified by Co_0.5_Mn_0.5_Fe_2_O_4_ in Ratio 80/20 on the Expression of Genes Related to microRNA Involved in the Process of Osteoblastogenesis and Osteoclatogenesis towards Pre-Osteoblasts and Pre-Osteoclasts in the Presence of Magnetic Field

**Figure 11:**
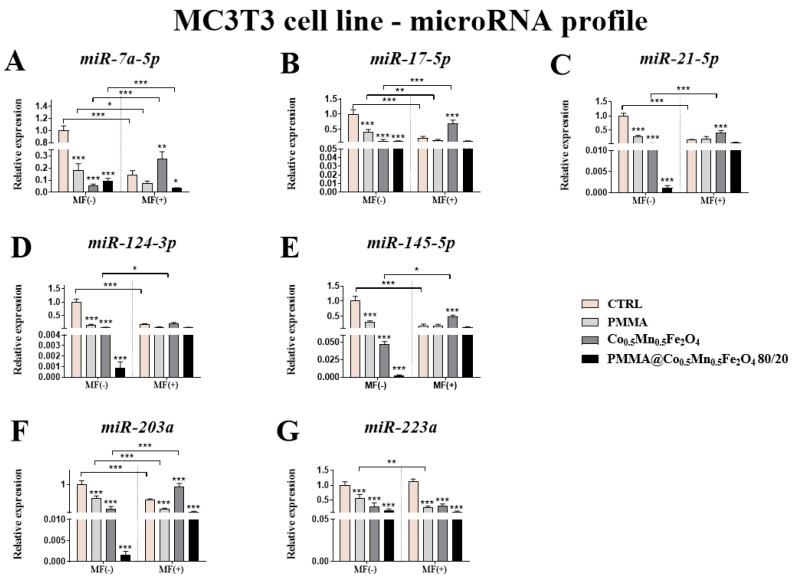
The impact of PMMA and its modification on the microRNA profile associated with osteoblastogenesis and osteoclastogenesis expression of *miR-7a-5p* (**A**), *miR-17-5p* (**B**), *miR-21-5p* (**C**), *miR-124-3p* (**D**), *miR-145-5p* (**E**), *miR-203a* (**F**) and *miR-223a* (**G**) in the magnetic field condition towards mouse pre-osteoblasts (MC3T3-E1 cell line. Statistical differences are indicated by * *p* < 0.005; ** *p* < 0.001 and *** *p* < 0.0001.

Additional studies revealed statistically significant decrease of the expression of *miR-17-5p, miR-21-5p, miR-124-3p* and *miR-145-5p* in comparison to control only in case of MF absence, while the expression of *miR-7a-5p, miR-203a* and *miR-223a* was decreased independently of the magnetic field presence in the MC3T3-E1 cell line (Figure 11A–G).

Comparing the results between the presence and absence of the magnetic field, only in the case of *miR-7a-5p* was the slightly decreased of expression of this miR observed under magnetic field condition (Figure 11A).

**Figure 12:**
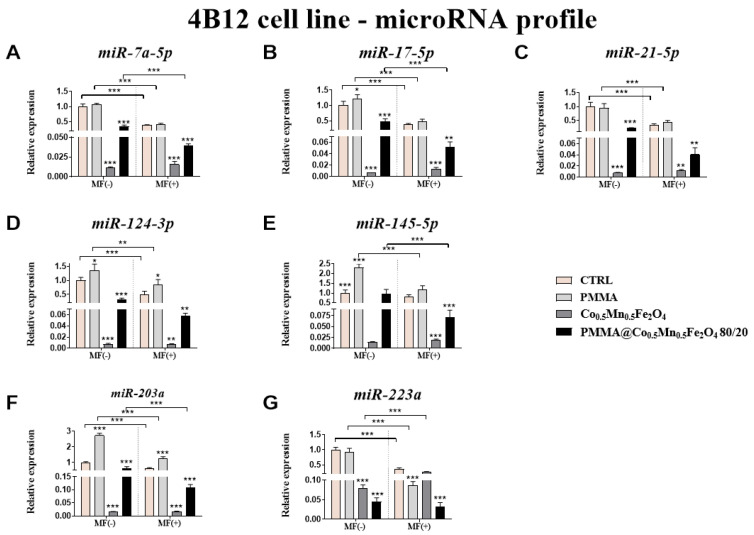
The impact of PMMA and its modification on the microRNA profile associated with osteoblastogenesis and osteoclastogenesis expression of *miR-7a-5p* (**A**), *miR-17-5p* (**B**), *miR-21-5p* (**C**), *miR-124-3p* (**D**), *miR-145-5p* (**E**), *miR-203a* (**F**) and *miR-223a* (**G**) in the magnetic field condition towards mouse pre-osteoclasts (4B12 cell line). Statistical differences are indicated by * *p* < 0.005; ** *p* < 0.001 and *** *p* < 0.0001.

From the other side, in pre-osteoclasts (4B12 cell line) almost all tested microRNA (*miR-7a-5p*, *miR-17-5p*, *miR-21-5p*, *miR124-3p*, *miR-203a* and *miR-223a*) was decreased after incubation with Co_0.5_Mn_0.5_Fe_2_O_4_@PMMA in ratio 80/20 independently of the presence of the magnetic field (Figure 12A–D,F,G). However, the expression of *miR-145-5p* was diminished only in the presence of magnetic field (Figure 12E). It was interesting that MF caused strongest decrease of the expression of *miR-7a-5p*, *miR-17-5p*, *miR-145-5p*, *miR-203a* and *miR-223a*, comparing to the results obtained in case of MF absence (Figure 12A–C,E–G).

## 4. Discussion

The effectiveness of bone implants tailored to osteoporotic patients depends on both stimulation of osteoblast proliferation and differentiation, as well as inhibition of osteoclasts activity. Poly(methyl methacrylate) (PMMA) has become one of the attractive and frequently used polymers in the synthesis of bone cements since its first biomedical application in 1937. In order to enhance its bioactivity, PMMA can be combined with a wide range of chemicals in order to synthetize nanoparticles with improved osteoinductive properties. One of the potential candidates for small molecule additives are bioinorganic ions which represents an inexpensive and stable alternative to peptides, nucleic acids and growth factors. In the presented study, we doped PMMA with Co, Mn and Fe_2_O_4_ and analyzed in vitro the osteogenic and osteoclastogenic properties of composite. Furthermore, we have investigated whether exposition of fabricated biomaterials to magnetic field enhance their bioactive properties. Previous studies have confirmed that cobalt ions and MF are both able to enhance angiogenesis and bone tissue regeneration, which supports their application in the fabrication of nanomaterials for bone tissue engineering. 

For that reason, in order to confirm the utility of bone-filling material, its impact on the cells surrounding the microenvironment of damaged tissue should be investigated carefully. Biocompatibility results revealed that pure Co decreased proliferation of MC3T3-E1 cell line in comparison to control group; however, that effect was ameliorated in PMMA + Co group. Previous studies revealed that a concentration of cobalt ions <10 ppm enhance proliferation of bone cells [23,24]. In the presented study, we employed the MC3T3-E1 cell line as it has behaviors similar to primary calvarial osteoblasts and for the evaluation of biocompatibility of implants is preferable to osteosarcoma cell line, since it better reflects physiological condition. A similar phenomenon was observed for 4B12 cells, in which addition of Co resulted in decreased growth kinetics.

In the presented study, we demonstrated that Co_0.5_Mn_0.5_Fe_2_O_4_@PMMA hybrid and Co_0.5_Mn_0.5_Fe_2_O_4_ alone exert a pro-ostegoenic and anit-osteoclastogenic effects. The biological activity of the materials has been demonstrated by the analysis of gene expression related to bone cells metabolism under normal and MF condition. Interestingly, hybrid material showed the better cell response for the expression of *Runx2*, *Alp*, *Col1a1*, while Co_0.5_Mn_0.5_Fe_2_O_4_ enhanced expression of *Opn*, *Bglap2*, *Dmp1*. Observed phenomenon can be explained at least by two different ways. Enhanced differentiation of MC3T3-E1 cells may by directly activated by each hybrid components. Modification of PMMA surface with different inorganic compounds was proved to enhance osteoblasts adhesion and response. Recently, it was shown by Phakatkar et al. [25] that novel PMMA bone cement nanocomposites containing magnesium phosphate nanosheets and hydroxyapatite nanofibers possess antibacterial attributes with enhanced cytocompatibility and mechanical properties. Another group showed that incorporation of hydroxyapatite into PMMA increases the biological response to the cement from tissue around the implant site [20]. The authors revealed that, after the transplantation of material in vivo, its surface is immediately covered by the cells with initiate the osteointegration. They proved that the incorporation of hydroxyapatite improves the attachment of extracellular matrix (ECM) protein in comparison to PMMA only. In the same analogy, enhanced osteogenesis on the PMMA hybrid observed in the presented study may results from its modification with inorganic ions. Fan et al. [23] have shown that cobalt chloride (CoCl_2_-treated bone progenitor cells induced higher degree of vascularization and enhanced osteogenesis. Furthermore, cobalt-substituted hydroxyapatite (COHA) effectively promotes bone cell growth, reduces the inflammatory response and is an antibacterial agent [26].

Another possible mechanism is related to the presence of iron oxide in fabricated materials. Recently, iron oxide nanoparticles (IONPs) have been widely studied in the areas of bone regenerative medicine. It was shown that IONPs incorporated to gelatin sponge scaffold enhance bone formation in vivo and is visible in MRI imaging without using of external magnetic field [27]. On the other hand, nanocomposites of iron oxide and hydroxyapatite were characterized by superparamagnetic and biocompatible properties [28]. Vlad et al. [27] have shown that incorporation of iron oxide nanoparticles into the powder phase of an alpha-tricalcium phosphate-based cement improved injectability of apatitic bone cement for vertebroplasty. The application of IONPs in bone tissue engineering was also investigated in our own studies. We have shown previously that α-Fe_2_O_3_/γ-Fe_2_O_3_ nanocomposite exerts dual action as they enhance osteogenic differentiation while reduce the activity of osteoclast [20]. We also found that polyurethane/poly(lactic) acid sponges doped with iron oxide nanoparticles under magnetic field enhance osteogenesis of adipose derived mesenchymal stem cells by enhanced expression of osteopontin and collagen type I.

An additional mechanism for better cell response may result from the application of external magnetic field (MF), which is therapeutic agent per se and further enhance the bioactive properties of materials doped with magnetic responsive particles. MF potential to affect osteoblast behavior on different biomaterials have been proved in multiple studies including our own. Based on the obtained data, MF represent a potential tool to improve the clinical outcome of selected regenerative therapies not only in orthopedics but also in dentistry. However, due to discrepancies between some research works, MF should be more thoroughly investigated by proper clinical trials. Herein, we have shown that application of MF enhances the cellular response of scaffold doped with Fe_2_O_4_. It stands with our and other previous research which showed that MF application enhance osteogenesis, modulates progenitor stem cells fate and diminish osteoclasts activity. 

It also should be mentioned that fabricated, hybrid material, due to the incorporation of Co and iron oxides, represents a potent MRI contrasting agent. In diagnostics, MRI is applied to differ between healthy and tumor tissue and to visualize the location of lesions. However, metal implants can interfere with the MRI, causing misinterpretation of the obtained results. In previous experiments, it was confirmed that COHAC can be used as a T2 contrast localization agent and does not cause image interference [29,30].

In the next step, we investigated how fabricated nanocomposites affects the expression of the integrins as they are involved in the regulation of multiple cell functions, including their interactions with matrix, proliferation and differentiation. It was shown that cell movement on the material surface is possible through the formation of cytoskeletal projections called filopodia, which in turn stimulates the activation of integrins [18,31]. Transmembrane receptors in a great amount can be found in cells forming focal contacts (adhesion plaques), which are directly responsible for the adhesion of cells to material surface. Integrins bound to selected ECM components, e.g., collagen, fibronectin, osteopontin and through signal transduction modulates the fate of bone forming cells and, thus, are directly involved in the regeneration process. Their activation stimulates cells to migration, movement, adhesion and, finally, differentiation. The findings in this study show that both Co_0.5_Mn_0.5_Fe_2_O_4_@PMMA and Co_0.5_Mn_0.5_Fe_2_O_4_ in control and MF condition modulate the integrins expression. We found that the abovementioned materials enhanced the mRNA levels of *INTa6*, *INTa1* and *INTa3* in relation to pure PMMA. 

Here, we also proved that the new biomaterials could influence on the action of miRNAs involved in the processes of bone remodeling and can modulate the pro-inflammatory response that is important in the case of the potential future use of such biomaterials in the OP treatments.

## 5. Conclusions

In the presented paper, we fabricated a Co_0.5_Mn_0.5_Fe_2_O_4_@PMMA hybrid and investigated its physicochemical and biological properties. The bioactivity of scaffolds was determined using in vitro osteoblast and osteoclasts system. The hybrid material showed better cellular response in comparison to pure PMMA under control and magnetic field condition. This can be explained by the presence of bioactive, inorganic ions- Fe_2_O_4_ and Co, which are known to enhance bone regeneration. Therefore, a newly developed Co_0.5_Mn_0.5_Fe_2_O_4_@PMMA might be useful for a bone substitute or filler.

The material for bone regeneration should be characterized by activation of preosteoblasts, which induce deposition of ECM proteins and leads to its mineralization. Yet, while taking into consideration its application in osteoporotic patients, novel, smart biomaterials should be incorporated with particles that not only stimulate bone forming cells, but at the same time inhibit the overactivity of osteoclasts. The enhanced activity of bone resorbing cells disrupts tissue homeostasis, contributing to bone mass loss and altered bone microstructures, which make it prone to fractures. Thus, in order to re-establish normal bone repair, bone grafts should be tailored to meet the patients’ need and restore the balance between cells in affected tissue. Here, we provide a proof of evidence that the modification of PMMA with Co_0.5_Mn_0.5_Fe_2_O may represent a novel approach for the material optimization and may be the way forward in the fabrication of scaffold with enhanced bioactivity that benefits osteoporotic patients. 

## Figures and Tables

**Table 1:** Sequences of primers used in the actual studies.

Gene	Forward (5′→3′)	Reverse (3′→5′)	Length of Amplicon
P21	TGTTCCACACAGGAGCAAAG	AACACGCTCCCAGACGTAGT	175
P53	AGTCACAGCACATGACGGAGG	GGAGTCTTCCAGTGTGATGATGG	287
CASP9	CCGGTGGACATTGGTTCTGG	GCCATCTCCATCAAAGCCGT	278
BAD	ACATTCATCAGCAGGGACGG	ATCCCTTCATCCTCCTCGGT	115
BAX	AGGACGCATCCACCAAGAAGC	GGTTCTGATCAGCTCGGGCA	251
BCL2	GGATCCAGGATAACGGAGGC	ATGCACCCAGAGTGATGCAG	141
RUNX2	TCCGAAATGCCTCTGCTGTT	GCCACTTGGGGAGGATTTGT	130
RANKL	TTAAGCCAGTGCTTCACGGG	ACGTAGACCACGATGATGTCGC	493
OPG	TGGCACACAGTGATGAATGCG	GCTGGAAAGTTTGCTCTTGCG	149
ALP	TTCATAAGCAGGCGGGGGAG	TGAGATTCGTCCCTCGCTGG	198
COL1A1	CCAGCCGCAAAGAGTCTACA	CAGGTTTCCACGTCTCACCA	175
OPN	AGACCATGCAGAGAGCGAG	GCCCTTTCCGTTGTTGTCCT	340
BGLAP2	CTCCTGAGAGTCTGACAAAGCCTT	GCTGTGACATCCATTACTTGC	320
DMP1	CCCAGAGGCACAGGCAAATA	TCCTCCCCAATGTCCTTCTT	211
MMP9	TTGCCCCTACTGGAAGGTATTAT	GAGAATCTCTGAGCAATCCTTGA	172
PU.1	GAGAAGCTGATGGCTTGGAG	TTGTGCTTGGACGAGAACTG	175
ITGAV	ACAATGTAAGCCCAGTTGTGTCT	TTTGTAAGGCCACTGGAGATTTA	236
C-FOS	CCAGTCAAGAGCATCAGCAA	TAAGTAGTGCAGCCCGGAGT	248
INTa1	CACCTTTCAAACTGAGCCCGCCA	GCTGCCCAGCGATGTAGAGCACAT	110
INTa3	TGGGCAAGTGCTATGTGCGTGGCA	TCTGGGTGAAGCCGCCGCTGGT	147
INTa6	CTGGCTTCCTCGTTTGGCTATG	TGCCTTGCTGGTTAATGTAGACGT	145
INTb1	TCTCACCAAAGTAGAAAGCAGGGA	ACGATAGCTTCATTGTTGCCATTC	138
IL6	GAGGATACCACTCCCAACAGACC	AAGTGCATCATCGTTGTTCATACA	141
TGFβ1	GGAGAGCCCTGGATACCAAC	CAACCCAGGTCCTTCCTAAA	171
TNFα	GAACTGGCAGAAGAGGCACT	AGGGTCTGGGCCATAGAACT	203
miR-7a-5p	TGGAAGACTAGTGATTTTGTTGT	*	
miR-17-5p	CAAAGTGCTTACAGTGCAGGTAG	*	
miR-145-5p	GTCCAGTTTTCCCAGGAATCCCT	*	
miR-21-5p	TAGCTTATCAGACTGATGTTGA	*	
miR-124-3p	TAAGGCACGCGGTGAATGCCAA	*	
miR-203a	GUGAAAUGUUUAGGACCACUAG	*	
miR-223a	TGTCAGTTTGTCAAATACCCCA	*	
GAPDH	TGCACCACCAACTGCTTAG	GGATGCAGGGATGATGTTC	177

*p21:* cyclin-dependent kinase inhibitor 1; *p53:* tumor suppressor factor; *Casp9:* caspase 9; *Bad:* Bcl-2 associated agonist of cell death; *Bax:* Bcl-2 associated X protein; *Bcl-2:* B-cell lymphoma 2; *RUNX-2:* Runt-related transcription factor 2; *RANKL:* Receptor Activator for Nucleat Factor κβ Ligand; *Opg:* osteoprotegerin; *Alp:* phosphatase alkaline; *Col1A1:* collagen alpha-1 chain precursor; *Opn:* osteoponin; *Bglap2:* bone-carboxyglutamic acid-containing protein; *Dmp-1:* dentin matrix protein 1; *Mmp-9:* matrix metalloproteinase 9; *PU.1:* protein in human encoded by the SPI1 gene; *Itgav:* Integrin Subunit Alpha V; *c-fos:* protoonkogene, cellular oncogene fos; *INTa1:* intergrin alpha 1; *INTa3:* integrin alpha 3; *INTa6:* integrin alpha 6; *INTb1*: integrin beta 1; *Il-6:* interleukin-6, *Tgfβ1:* tumor-growth factor beta 1; *Tnfa;* tumor-necrosis factor alpha, *miR*; microRNA, *GAPDH:* Glyceraldehyde-3-phosphate dehydrogenase.* there is only one sequence.

## Data Availability

The data presented in this study are available on request from the corresponding author. The data are not publicly available due to ongoing studies.

## Abbreviations

ALP	alkaline phosphatase
BAD	Bcl-2 associated agonist of cell death
BAX	Bcl-2 associated X protein
Bcl-2	B-cell lymphoma 2
BGLAP	bone gamma-carboxyglutamic acid-containing protein (OC)
BMD	bone mineral density
CASP	caspase
c-FOS	cellular protooncogene
COHA	cobalt-substitute hydroxyapatitie
COL1A1	Collagen alpha-1 (I) chain precursor
CSF1	Colony stimulating factor 1
CSCM	30% calvaria-derived stromal cell conditioned media
DEPC	sterile filter water treated with diethyl pyrocarbonate
DMP1	dentin matrix acidic phosphoprotein 1
ECM	extracellular matrix
FBS	Fetal Bovine Serum
FTIR-ATR	Fourier Transform Infrared-Attenuated Total Reflectance
GAPDH	glyceraldehyde 3-phosphate dehydrogenase
IL	interleukin
IONs	iron oxide nanoparticles
ITGAV	Integrin Subunit AlphaV
iNOS	inducible nitric oxide synthase
INT	integrin
MEMα	Minimum Essential Medium α
miRNA	microRNA
MF	magnetic field
MMP	matrix metalloproteinase
MNPs	magnetic nanoparticles
OC	osteocalcin (BGLAP)
OPN	osteopontin
OS	osteoporosis
p21	cyclin-dependent kinase inhibitor 1
p53	tumor suppressor factor
PMMA	polymethylcrylate
PU.1	protein in human encoded by the SPI1 gene
RANKL	Receptor activator of NFκβ ligand
RUNX2	Runt-related transcription factor 2
SAED	selected area (electron) diffraction
SEM	scanning electron microscopy
SMF	static magnetic field
TEM	transmission electron microscopy
TNFα	tumor necrosis factor α
TGFβ	transforming growth factor β
